# Association between local-level resources for home care and home deaths: A nationwide spatial analysis in Japan

**DOI:** 10.1371/journal.pone.0201649

**Published:** 2018-08-24

**Authors:** Noriko Morioka, Jun Tomio, Toshikazu Seto, Yoshie Yumoto, Yasuko Ogata, Yasuki Kobayashi

**Affiliations:** 1 Department of Gerontological Nursing & Care System Development, Tokyo Medical and Dental University, Tokyo, Japan; 2 Department of Public Health, Graduate School of Medicine, The University of Tokyo, Tokyo, Japan; 3 Center for Spatial Information Science, The University of Tokyo, Tokyo, Japan; Hamamatsu Ika Daigaku, JAPAN

## Abstract

**Aim:**

Little is known about whether and how local-level resources regarding home care are associated with the prevalence of home deaths. We aimed to investigate whether geographic patterns of the resources for home care were associated with the prevalence of home deaths, taking spatial variation into consideration.

**Methods:**

We conducted an ecological cross-sectional study in Japan using nationwide data in 2014. The areal unit was the municipality, the smallest administrative unit in Japan. We investigated the association between the percentage of home deaths and the resources of home care support clinics with available 24-hour-a-day functions, considering the geographic effect of neighboring municipalities by applying a geographically weighted regression model.

**Results:**

The mean and standard deviation of the percentages of home deaths were 11.4% (5.0%), and those of the number of home care support clinics per 10,000 elderly population were 3.4 (3.7). The percentages of home deaths in neighboring municipalities tended to be significantly correlated (Moran’s I 0.34, p<0.001). Adjusting for the number of hospital beds, total population, and the socio-economic status of municipality, the results of an ordinary least squares regression model showed a positive correlation between the percentage of home deaths and the local resources for home care support clinics per 10,000 elderly population (regression coefficient 0.15, 95% confidence interval 0.07, 0.22), while the existence of spatial autocorrelation of the residual was suggested (Moran’s I of the residual 0.227, p<0.001). The geographically weighted regression model showed local regression coefficients varying across municipalities with a better model fit over the analogous ordinary least squares model (adjusted R^2^ 0.414 vs. 0.131).

**Conclusion:**

Home deaths were more prevalent in municipalities with greater home care resources. This association was geographically varied and further strengthened in some areas.

## Introduction

Place of death is a matter of concern in an aging society. Ensuring that older persons receive appropriate end-of-life (EOL) care at home is an urgent issue in many aging societies [1,2]. Although over 50% of people prefer to receive their EOL care at home [3], only 12.7% died at home in Japan [4]. There is an imbalance between preference and actual place of death, as seen in cases in the United States and in European countries [3,5–7].

Home death depends on multiple factors. In addition to individual factors such as socioeconomic characteristics and illness-related factors, regional factors such as rural/urban setting, deprived area or not, and home-based medical service resources were associated with home deaths [8–11]. Previous ecological studies in Japan showed that higher percentages of home deaths were associated with higher resources for home-based medical care by prefecture (state) level [12–14]. However, analyses performed at the prefecture level might be too large in terms of areas to reveal the underlying spatial pattern [15]. To better understand the association between home deaths and home-based medical care resources, geographic analysis at a local level is necessary.

Moreover, no studies have considered geographical effects in their analyses. Recent studies in public health have taken into account geographical effects of the neighboring environment to investigate the association between health outcomes and risk factors [16–19]. Regional socioeconomic status often exhibits spatial autocorrelation, where neighboring observation values tend to be correlated, and where relationships between variables may differ depending on the location [16,17,20]. When investigating the association between home deaths and resources of home care at the municipality level, it is important to understand the geographical effects of neighboring municipalities.

We, therefore, aimed to investigate the association between the percentage of home deaths and the resources of home-based medical care at the municipality level, considering the geographic effects of neighboring municipalities using spatial analysis.

## Materials and methods

### Setting

We conducted an ecological cross-sectional study using nationwide data in Japan. The areal unit was the municipality, which is the smallest unit of an administrative district in Japan. There were 1,741 municipalities in 47 prefectures as of 2014.

### Home medical care in Japan

In Japan, all citizens have medical care and long-term care coverage under universal health insurance, such as employee-based Health Insurance, National Health Insurance for self-employed people and pensioners under 75 years of age, and Late Elders’ Health Insurance [21,22]. In addition, Long-Term Care Insurance (LTCI) was introduced in 2000, in which the insurers are the municipalities [23]. Home-based medical care services are covered by health insurance. In 2006, a revision of the medical fee schedule introduced home care support clinics (HCSCs) with home care support functions available 24-hour a day until the patient dies. HCSCs should have a system in place enabling 24-hour home visit care and/or home visit nursing at the patient’s request (the conventional requirements) [24]. In 2012, enhanced HCSCs were institutionalized. Enhanced HCSCs should meet the following requirements, which were added to the conventional requirements: three or more full-time doctors appointed, five or more cases of emergency home visits in the past year, and two or more cases of EOL care in the past year. If an HCSC meets the enhanced HCSC requirements, the HCSC can obtain a higher fee than that of an HCSC that only meets the conventional requirements [24].

While home-based medical care services are covered by health insurance, home-visit nursing care services are covered by both health insurance and LTCI, depending on the illness and/or age of a person. With an increasing elderly population (aged 65 and over), the government has promoted the “integrated community care system,” that is, a system that “enables citizens to keep living in a familiar environment, regardless of the type of housing, through the use of various services provided locally, around the clock and 365 days a year” [25]. Ensuring high-quality home medical care and home-visit nursing care is a key element to achieving an integrated community care system [22].

### Data source

We obtained our dataset from Japan’s Ministry of Health, Labour and Welfare (MHLW) via its website [26]. The MHLW re-aggregated Vital Statistics in 2014 [27], and obtained data from the Survey of Medical Institutions in 2014 [28] and Survey of Institutions and Establishments for Long-term Care in 2014 [29] by municipalities, disclosing the dataset on the website on July 10, 2015. The dataset includes variables at the municipality level, including the percentage of home deaths (per total deaths), the number of HCSCs, the number of home-visiting nurse agencies, the total population, and the elderly population. We also obtained the socioeconomic status of municipalities from the Social Observations of Shi, Ku, Machi, and Mura (municipalities) in 2014 [30]. We obtained the number of hospital beds from Medical Institution Survey [28]. This study exclusively used published data and did not handle any personal data.

### Variables

#### Home care resources

As resources for home medical and nursing care include 24-hour support through EOL at home, we used the number of HCSCs per 10,000 elderly population in each municipality.

#### Percentage of home deaths

The percentage of home deaths was the number of deaths at home out of the total number of deaths in 2014. Because the place of death (home, hospital, care facilities, and so on) was indicated in the data, home deaths included deaths when the patient did not receive home-based medical care, such as sudden deaths at home.

#### Medical and long-term care resources and socioeconomic variables

Medical and long-term care resources and socioeconomic variables associated with deaths at home were selected based on a review of the literature [12,13,31–33] and available data. Previous studies suggested that deaths at home were due to poor hospital and medical facility resources [12,13]. We used the number of hospital beds per 10,000 elderly population, as well as the number of beds of long-term care insurance facilities per 10,000 elderly population. Not living alone was also a determinant of death at home [13,31–33]. As a proxy for living alone, we used the percentage of single-person households with elderly people by calculating the number of one-person households with elderly divided by the total number of households with elderly people. We also used the total population, percentage of elderly people, and the average per capita annual income.

### Statistical and spatial analysis

#### Detection of spatial autocorrelation

Spatial autocorrelation in the percentage of home deaths was evaluated using a global Moran’s I test [34]. The Moran’s I test measures the degree to which disease pattern is clustered, dispersed, or randomly distributed across municipality tracts by computing the deviation from the mean for each tract. Moran’s I value ranges from -1 (perfect dispersion) to 1 (perfect clustering), and zero corresponds to a random spatial pattern.

#### Ordinary least square (OLS) regression

In the regression analysis, we included 1,718 municipalities excluding 23 municipalities where the percentages of home death were non-disclosed. A separate variance t-test showed that the means of the total population in 23 municipalities were significantly lower (p<0.001) and the means of the percentage of elderly people in the total population in 23 municipalities were significantly higher (p<0.001) than those in 1,718 municipalities, respectively.

Due to heteroskedasticity, we applied a robust OLS regression model [35] to investigate the association between the percentage of home deaths and capacity of home care, adjusting for the number of hospital beds per 10,000 elderly population, the number of beds of long-term care insurance facilities per 10,000 elderly population, the total population, the percentage of elderly people, the average per capita annual income, percentage of elderly population, and the percentage of single-person households with elderly people as covariates. To determine whether the independent identically distributed assumptions of OLS regression were being met, we conducted Moran’s I test [34] for the residuals of the OLS model. When spatial autocorrelation of the residual is present, the use of a technique specifically designed for dealing with this type of problem, such as a geographically weighted regression model, is recommended [36]. We checked that our data met the assumptions of the OLS using a Pearson’s correlation (S1 Table) and the variance inflation factor (<5).

#### Geographically weighted regression (GWR)

The next step was to build a GWR model that included the variables in the OLS model. GWR extends the OLS framework as follows:

$$y_{i}=\beta_{0}\left(u_{i},v_{i}\right)+\sum_{k}\beta_{k}\left(u_{i},v_{i}\right)x_{ik}+\varepsilon_{i}$$

where (*u*_*i*_, *v*_*i*_) denotes the coordinates of the *i*th point in space, *β*_*k*_(*u*_*i*_, *v*_*i*_) is a realization of the continuous function *β*_*k*_(*u*, *v*) at point *i*, and *ε*_*i*_ is the *i*th residual [36]. A Gaussian linear model with an adaptive bi-square geographic kernel was used assigning a weight to each observation. We used the “golden section search” function for optimal bandwidth selection by minimizing Akaike corrected Information Criterion (AICc) as the criterion, with a lower AICc indicating improvement of model fitness [36,37]. To assess the goodness of fit between the OLS and GWR models, the R^2^ of the OLS model was compared to the R^2^ of a GWR with a higher R^2^ indicating greater explanation of variance in the model. In addition to the R^2^, the AICc and Moran’s I statistic of the residuals were used to compare the two model types. The details of the statistical profile of the GWR are described elsewhere [36]. For a subgroup analysis, we conducted the same statistical models with the two types of HCSC mentioned above, the enhanced type and the conventional type.

The p-value <0.05 was considered to be statistically significant. Choropleth maps and Moran’s I statistics were generated using ArcGIS (Esri), version 10.4. GWR analyses were conducted in GWR, version 4.0 [37]. For all other statistical analyses, we used Stata (StataCorp), version 13.1.

## Results

The mean and standard deviation of the percentages of home deaths were 11.4% (5.0%), and those of the number of HCSCs per 10,000 elderly population were 3.4 (3.7) (Table 1).

**Table 1:** Characteristics of the 1,741 Municipalities in Japan.

Variable	Mean	SD	Min	Max
The percentage of home deaths (%)^a^	11.4	5.0	0.9	54.8
The number of HCSCs per 10,000 elderly population				
Total	3.4	3.7	0.0	35.5
Enhanced HCSCs	0.8	1.7	0.0	21.0
Conventional HCSCs	2.6	3.2	0.0	35.5
The number of hospital beds per 10,000 elderly population	3.8	3.6	0.0	39.2
The number of beds of long-term care facilities per 10,000 elderly population	3.6	2.3	0.0	27.9
Total population (10,000 persons)	7.3	18.2	0.0	363.9
Average per capita annual income (million yen)	2.8	0.5	1.9	9.0
Percentage of elderly people (%)	29.7	6.7	13.1	58.0
Percentage of single-person households with elderly people (%)^b^	22.0	7.4	5.6	60.0

p<0.001, EOL: end-of-life; HCSC: home care support clinics; SD: standard deviation, elderly: aged 65 or over.

^a^ Data in 23 out of 1,741 municipalities were missing.

^b^ Percentage of single-person households with elderly people (%) calculated by the number of one-person households with elderly divided by the total number of households with elderly people.

**Fig 1:**
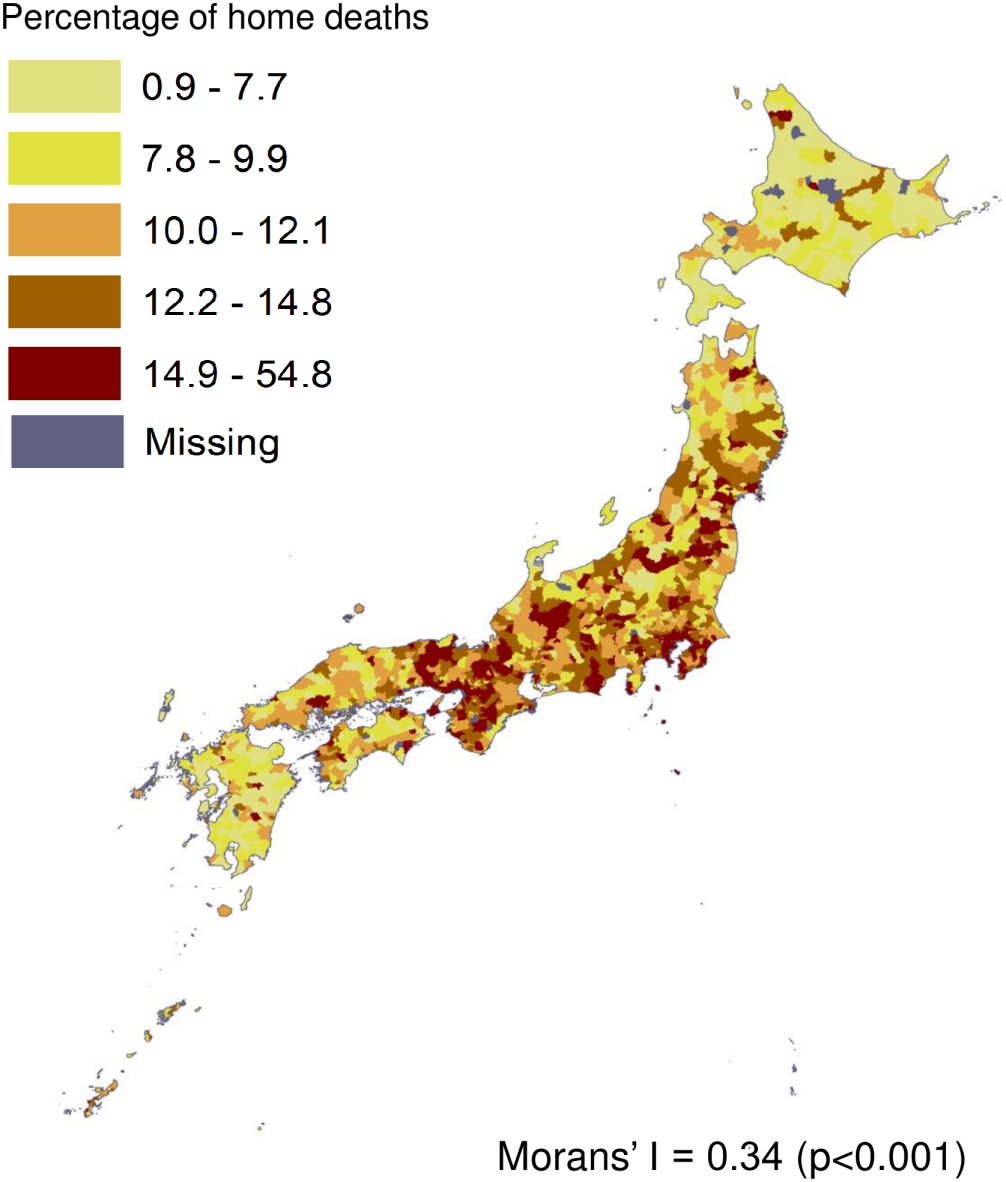
Geographic distribution of the percentage of Home Deaths in 1,741 Municipalities in Japan. A Moran’s I value ranges from -1 (perfect dispersion) to 1 (perfect clustering), and zero corresponds to a random spatial pattern. The value of 0.34 suggests that the percentages of home deaths in municipalities were moderately spatially autocorrelated. The authors created the map based on the spatial vector data of municipalities obtained from the National Land Numerical Information download service (http://nlftp.mlit.go.jp/ksj-e/index.html), Ministry of Land, Infrastructure, Transport and Tourism.

**Fig 2:**
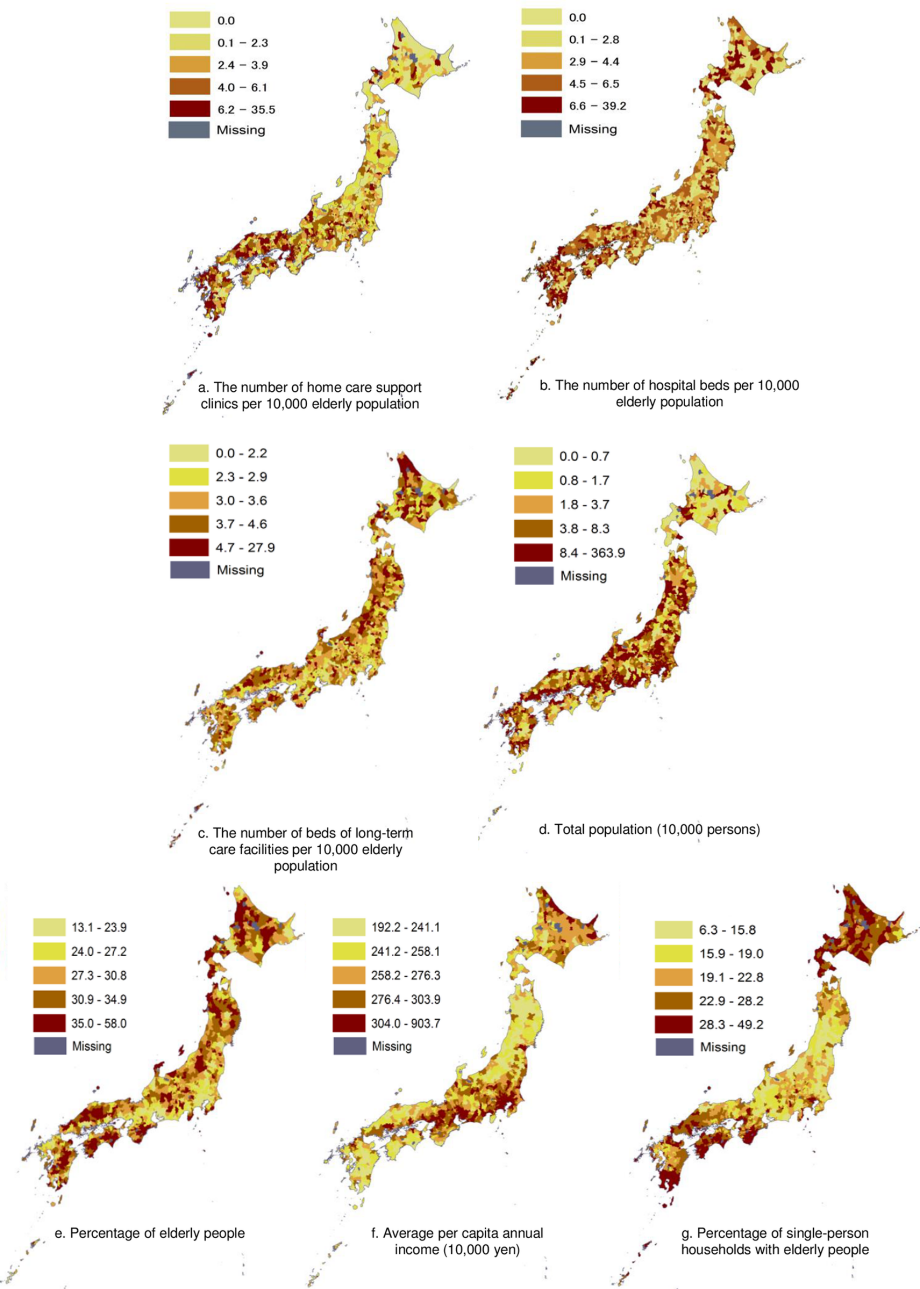
Geographic distribution of medical care resources and socioeconomic variables in 1,741 Municipalities in Japan. The authors created the maps based on spatial vector data of municipalities obtained from the National Land Numerical Information download service (http://nlftp.mlit.go.jp/ksj-e/index.html), Ministry of Land, Infrastructure, Transport and Tourism.

Fig 1 visually shows that the percentage of home deaths in each municipality varied widely from 0.9% to 54.8% and a moderate spatial autocorrelation was present (Moran’s I 0.34, p<0.001). The percentages of home deaths were likely to be higher in the central area of Japan and lower in the north area and the south west area. Fig 2 indicates that the number of HCSCs per 10,000 elderly population varied widely from 0.0 to 35.5, and approximately one fifth of the 1,741 municipalities had no HCSCs.

Table 2 shows a comparison between the results of the OLS and those of the GWR. The results of the OLS regression model show that an increase of one in the number of HCSC per 10,000 elderly population was associated with a 0.15% increase of the percentage of home deaths (95% CI 0.07% to 0.22%). The results of the GWR model show that the local coefficients of the number of HCSCs per 10,000 elderly population varied across municipalities (median 0.16, inter-quartile range 0.08 to 0.24), adjusting for the values in neighboring municipalities (Table 2, S1 Fig).

**Table 2:** Comparison results between the OLS and the GWR for the percentage of home deaths.

Variables	Global coefficients of OLS^a^				Local coefficients of GWR^b^				
	Coefficient	95% CI		P value	Mean	SD	25%tile	Median	75%tile
The number of HCSCs per 10,000 elderly population	0.15	0.07	0.22	<0.001	0.16	0.18	0.08	0.16	0.24
The number of hospital beds per 10,000 elderly population (100 beds)	-0.15	-0.23	-0.08	<0.001	-0.09	0.15	-0.18	-0.07	-0.01
The number of beds in long-term care facilities per 10,000 elderly population (100 beds)	-0.40	-0.54	-0.26	<0.001	-0.45	0.41	-0.61	-0.44	-0.25
Total population (10,000 persons)	0.02	0.01	0.03	<0.01	0.01	0.04	0.00	0.01	0.02
Average per capita annual income (million yen)	0.02	0.02	0.03	<0.001	0.01	0.03	-0.01	0.00	0.02
Percentage of elderly people (%)	0.03	-0.03	0.10	0.312	-0.01	0.17	-0.12	-0.04	0.09
Percentage of single-person households with elderly people (%)^c^	-0.06	-0.11	-0.02	<0.01	0.01	0.17	-0.12	0.02	0.14
Intercept	6.65	3.47	9.84	<0.001	11.25	9.97	6.25	11.91	16.04
Adjusted R^2^	0.131				0.414				
AICc	10197.5				9686.2				
Moran’s I of residuals^d^	0.227	p<0.001			0.041	p<0.05			

n = 1,718

AICc: Akaike corrected information criterion; CI: confidence interval; EOL: end-of-life; GWR: geographically weighted regression model; HCSC: home care support clinic; OLS: ordinary least square regression model; SD: standard deviation; Elderly refers to those aged 65 or over.

^a^ OLS with robust standard error

^b^ GWR settings

Model type: Gaussian; Geographic kernel: adaptive bi-square; Method for optimal bandwidth search: Golden section search; Criterion for optimal bandwidth: AICc; Best bandwidth size: 189.

^c^ Percentage of single-person households with elderly people (%) was found by calculating the number of one-person households with elderly people divided by the total number of households with elderly people.

^d^ Moran’s I: 42 municipalities that had no neighbors were excluded.

The associations that were found to be statistically significant in the model were between the percentage of home deaths and the number of hospital beds per 10,000 elderly population (β -0.15, 95% CI -0.23 to -0.08), the number of beds in long-term facilities per 10,000 elderly population (β -0.40, 95% CI -0.54 to -0.26), total population (β 0.02, 95% CI 0.01 to 0.03), the average per capita income (β 0.02, 95% CI 0.02 to 0.03) and the percentage of single-person households with elderly people (β -0.06, 95% CI -0.11 to -0.02). The percentage of elderly people was not statistically significant (Table 2).

**Fig 3:**
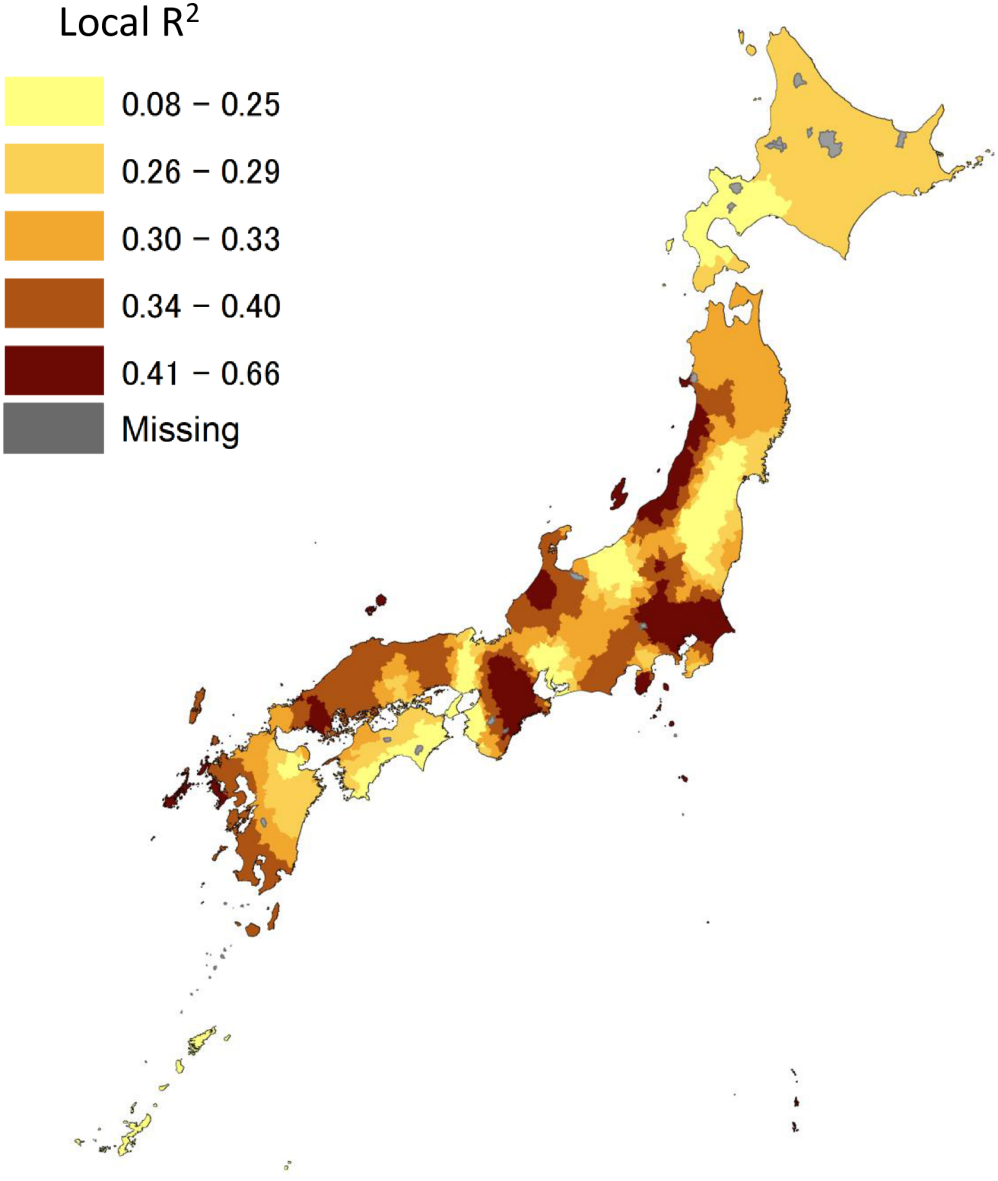
Distribution of the local adjusted R^2^ in 1,718 Municipalities in Japan in the GWR. The authors created the map based on spatial vector data of municipalities obtained from the National Land Numerical Information download service (http://nlftp.mlit.go.jp/ksj-e/index.html), Ministry of Land, Infrastructure, Transport and Tourism.

Moran’s I for the residuals in the OLS model was 0.227 (p<0.001), indicating that a weak but statistically significant spatial autocorrelation of the residual was present. Hence, Moran’s I for the residual in the GWR model was 0.041 (p<0.05), indicating that the residuals had an almost random pattern. Compared to the adjusted R^2^ and AICc in the OLS, those in the GWR improved from 0.131 to 0.414 and from 10197.5 to 9686.2, respectively. Fig 3 shows that the local R^2^ from GWR model ranged from 0.08 to 0.66, indicating that the GWR model can provide better estimates in some areas, such as the Tokyo metropolitan area, than in others.

In the subgroup analysis by two types of HCSCs, the associations between the higher numbers of each type of HCSC per 10,000 elderly population showed a statistically significant higher percentage of home deaths in the OLS model, but there are statistically significant spatial autocorrelations of the residuals. In the GWR models, the adjusted R^2^ and AICc improved more than those in the OLS (S2 Table).

## Discussion

To the best of our knowledge, this study was the first to investigate the associations between the percentage of home deaths and the resources of 24-hour home-based medical care at the municipality-level, considering the geographic effects of neighboring municipalities.

We found that municipalities with higher percentages of home deaths were likely to have greater resources of 24-hour home-based medical care and nursing care. In addition, this association was further strengthened in some areas, taking into account the resources in neighboring municipalities. This finding is consistent with those in previous studies [11,38]. A meta-analysis suggested that those receiving home-based EOL care [38] and home-based care by multidisciplinary teams with physicians, nurses, and other professionals were more likely to die at home [11]. To promote home death, ensuring the provision of 24-hour home care at the municipality level is crucial. An ecological study at the prefecture-level in Japan in 2010, however, reported that there was not a significant association between the number of HCSCs per total population and home death using univariate regression analysis [12]. There are several reasons that may explain the difference in results. First, and most importantly, the areal units are different. Second, the number of HCSCs increased from 12,500 in 2010 to 14,394 in 2014 [28]. The geographic distribution of HCSCs might have changed during those four years.

Importantly, our findings suggest that the association between home deaths and resources of HCSCs vary across municipalities. Considering neighboring environments using spatial analysis leads us to better understand the local trend in home deaths, especially in the Tokyo metropolitan area. As we expected, municipalities with higher percentages of home deaths were geographically clustered. Even in regions without enough HCSCs, if there are HCSCs in neighboring municipalities, there may be a higher possibility of dying at home. When constructing a 24-hour home-based care delivery system in a municipality, it is important to consider the medical and long-term care resources in neighboring municipalities.

We also found that the resources of hospitals and long-term care facilities were negatively associated with the percentage of home deaths, consistent with the findings of previous studies at the prefecture level [12,13]. During the past five decades, decreasing home deaths were related to an increasing number of hospital beds [13]. Differences in the resources for medical and/or long-term care may explain regional differences in the place of death [39]. The availability of long-term care and palliative care at a hospital may offset the probability of dying at home. A result is that death often happens in the hospitals in areas where there are sufficient hospitals and long-term care facilities; there might be less demand for home-based medical care.

Regarding socioeconomic status, a larger total population and higher average per capita annual income were associated with higher percentages of home deaths, adjusting for medical and long-term care resources. Cancer patients in lower socioeconomic groups were less likely both to die at home and to access home care [8]. People living in more deprived areas were more likely to die at a hospital, which might be caused by the inability to bear the costs of caring at home [40]. In Japan, the average cost of home-based medical care across 30 days was 1.32 times higher than the cost of hospitalization for EOL care (p = 0.3625, $266.8 vs. $202.0) [41]. In this context, the Japanese health care system, together with universal health insurance coverage, generally favors hospital care rather than home care. Individuals in areas with lower socioeconomic levels may be less likely to receive home-based medical care due to financial issues, even if there are clinics that provide home-based EOL care services. Improving access to home care for people in areas with lower socioeconomic levels is expected.

### Limitations

There are some limitations in this study. First, this is a cross-sectional study. We could not show a causal association between the percentage of home deaths and capacity of home care resources. Further studies using individual data of patients and facilities are necessary to investigate causality. Second, we excluded 1.3% (23) of the 1,741 municipalities. Such municipalities mostly included those with a small population and a high proportion of the elderly population, and this requires careful consideration in the interpretation of the results. Third, we might have overestimated the number of home deaths; because home deaths included deaths of people that did not receive any home-based care, such as sudden deaths and/or suicide at home, our data overestimated the number of people who received home-based care at EOL. We might also have overestimated the number of HCSCs that actually provided 24-hour home-based care, including EOL care, since we used the total number of HCSCs. However, subgroup analysis using only enhanced HCSCs provided similar results. Fourth, there might be unmeasured confounders such as patients’ and their families’ preferences, the number of users of home care, the number of physicians at clinics, accessibility, and other socio-economic status. Fifth, a modifiable areal unit problem is a geographic manifestation of the ecological fallacy in which conclusions based on data aggregated to a particular set of districts may change if one aggregates the same underlying data to a different set of districts. In other words, the way spatial data are aggregated may result in different findings [42]. Although our analysis used the smallest administrative district in Japan, the municipality level might still be too large to truly represent spatial differences in resources for home-based care and home death. Further investigation is necessary in this context.

## Conclusions

Greater resources for home care were significantly associated with a higher percentage of home deaths at the municipality level, and this association was further strengthened in some areas, which was associated with facilities in the neighboring environment. To enable elderly people to die at home in a familiar environment, it is necessary to promote home care delivery systems that are suitable for unique local conditions including taking neighboring municipalities into consideration.

## Supporting information

S1 Fig GWR of home care resources and the percentage of home deaths. Local coefficients of the number of home care support clinics 10,000 elderly population for the percentage of deaths at home in 1,718 Municipalities in Japan from the GWR model. The authors created the map based on spatial vector data of municipalities obtained from the National Land Numerical Information download service (http://nlftp.mlit.go.jp/ksj-e/index.html), Ministry of Land, Infrastructure, Transport and Tourism.
(TIF)

S1 Table Pearson’s coefficients among variables. (DOCX)

S2 Table Subgroup analysis for comparison results between the OLS and the GWR for the percentage of home deaths. (DOCX)
