# Supplementary figures and images for: Association between local-level resources for home care and home deaths: A nationwide spatial analysis in Japan
Source: PLoS One. 2018 Aug 24;13(8):e0201649. doi: 10.1371/journal.pone.0201649 (PMC6108466; doi:10.1371/journal.pone.0201649)

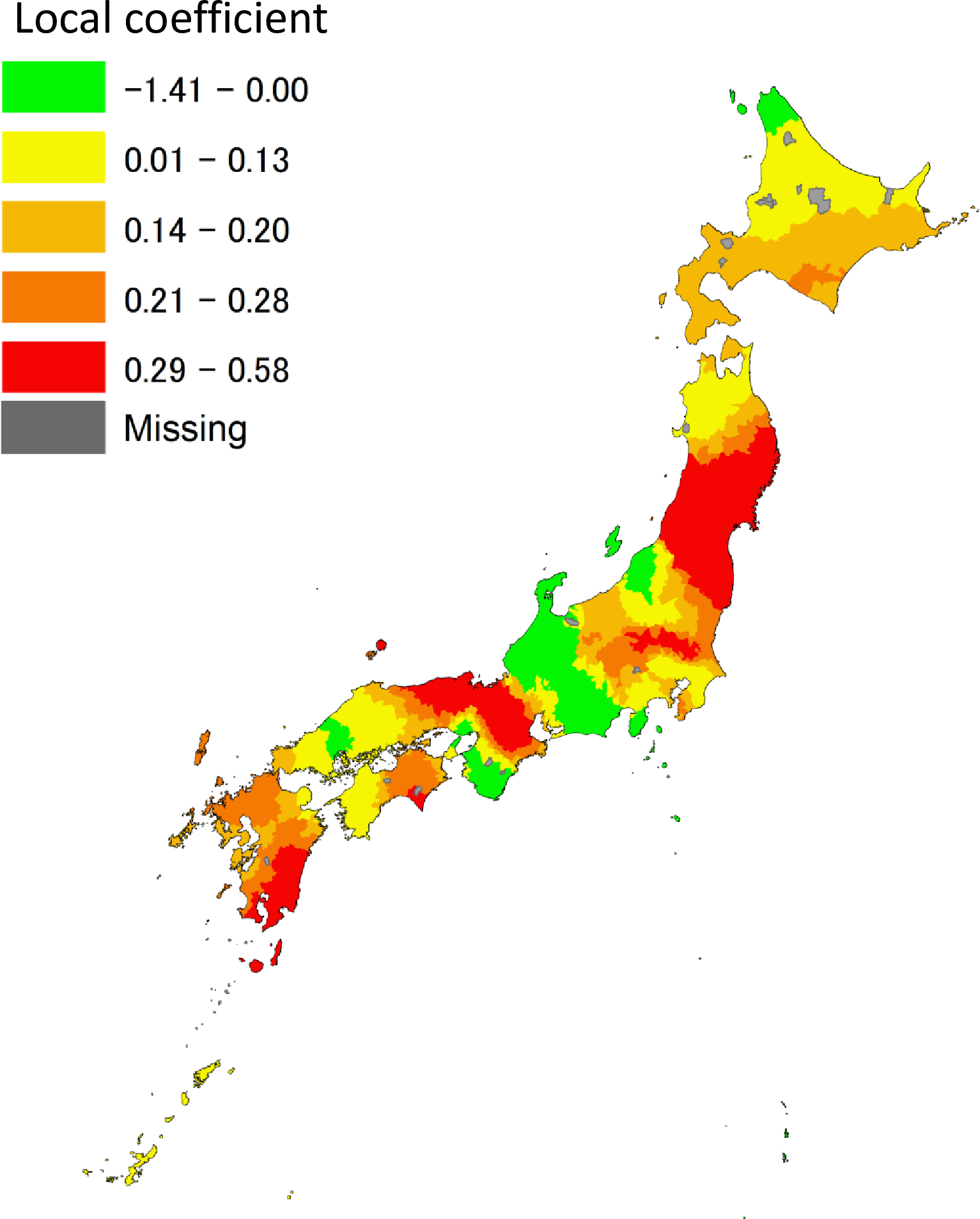

Supplement: S1 Fig — Local coefficients of the number of home care support clinics 10,000 elderly population for the percentage of deaths at home in 1,718 Municipalities in Japan from the GWR model. The authors created the map based on spatial vector data of municipalities obtained from the National Land Numerical Information download service (http://nlftp.mlit.go.jp/ksj-e/index.html), Ministry of Land, Infrastructure, Transport and Tourism. (TIF) [file pone.0201649.s001.tif]
