# Supplementary material for: Association between local-level resources for home care and home deaths: A nationwide spatial analysis in Japan
Source: PLoS One. 2018 Aug 24;13(8):e0201649. doi: 10.1371/journal.pone.0201649 (PMC6108466; doi:10.1371/journal.pone.0201649)
Supplement: S1 Table — (DOCX) [file pone.0201649.s002.docx]

|  | 1.The percentage of home deaths | | 2. The number of HCSCs per 10,000 elderly population | | 3. The number of hospital beds per 10,000 elderly population | | 4. The number of beds of long-term care facilities per 10,000 elderly population | | 5. Total population (10,000 persons) | | 6. Percentage of elderly people (%) | | | 7. Average per capita annual income (million yen) | | 8. Percentage of single-person households with elderly people (%) | | |
| --- | --- | --- | --- | --- | --- | --- | --- | --- | --- | --- | --- | --- | --- | --- | --- | --- | --- | --- |
| 1 | 1.000 |  |  |  |  |  |  |  |  |  |  |  |  | |  |  |  |  |
| 2 | 0.133 | ^***^ | 1.000 |  |  |  |  |  |  |  |  |  |  | |  |  |  |  |
| 3 | -0.110 | ^***^ | 0.078 | ^**^ | 1.000 |  |  |  |  |  |  |  |  | |  |  |  |  |
| 4 | -0.242 | ^***^ | 0.021 |  | 0.132 | ^***^ | 1.000 |  |  |  |  |  |  | |  |  |  |  |
| 5 | 0.137 | ^***^ | 0.138 | ^***^ | 0.130 | ^***^ | -0.126 | ^***^ | 1.000 |  |  |  |  | |  |  |  |  |
| 6 | -0.142 | ^***^ | -0.149 | ^***^ | -0.243 | ^***^ | 0.148 | ^***^ | -0.278 | ^***^ | 1.000 |  |  | |  |  |  |  |
| 7 | 0.255 | ^***^ | 0.200 | ^***^ | 0.142 | ^***^ | -0.202 | ^***^ | 0.347 | ^***^ | -0.555 | ^***^ | 1.000 | |  |  |  |  |
| 8 | -0.041 | ^*^ | 0.107 | ^***^ | 0.107 | ^***^ | -0.051 | ^**^ | 0.148 | ^***^ | 0.275 | ^***^ | 0.115 | | ^***^ | 1.000 |  |  |

HCSC: home care support clinics

Elderly means aged 65 or over.

* p<0.1, * p<0.05, *** p<0.001
