# Supplementary material for: Association between local-level resources for home care and home deaths: A nationwide spatial analysis in Japan
Source: PLoS One. 2018 Aug 24;13(8):e0201649. doi: 10.1371/journal.pone.0201649 (PMC6108466; doi:10.1371/journal.pone.0201649)
Supplement: S2 Table — (DOCX) [file pone.0201649.s003.docx]

| The number of HCSCs per 10,000 elderly population | OLS | | | | | Local coefficients of GWR^a^ | | | | | |
| --- | --- | --- | --- | --- | --- | --- | --- | --- | --- | --- | --- |
|  | Coefficient (95% CI) | P value | Adjusted R^2^ | AICc | Moran's I of residual^b^ | Mean(SD) | Median (IQR) | Bandwidth | Adjusted R^2^ | AICc | Moran's I of residual^b^ |
| Total | 0.15 (0.07, 0.22) | <0.001 | 0.131 | 10197.5 | 0.227^**^ | 0.16 (0.18) | 0.16 (0.08, 0.24) | 156 | 0.414 | 9686.2 | 0.041^*^ |
| Enhanced HCSCs | 0.30 (0.13, 0.47) | 0.001 | 0.133 | 10200.7 | 0.229^**^ | 0.25 (0.29) | 0.27 (0.10, 0.44) | 170 | 0.396 | 9720.2 | 0.040^*^ |
| Conventional HCSCs | 0.11 (0.03, 0.19) | 0.011 | 0.1286 | 10209.6 | 0.232^**^ | 0.12 (0.20) | 0.10 (0.00, 0.23) | 156 | 0.406 | 9709.3 | 0.042^*^ |

AICc: Akaike corrected Information Criterion; CI: confidence interval; GWR: Geographically weighted regression model; HCSC: home care support clinics; IQR: Interquartile Range; OLS: Ordinary least Square regression model; SD: Standard deviation

Elderly means aged 65 or over.

* p<0.05, ** p<0.001

Adjusted for the number of home-visiting nurse agencies per 10,000 elderly population, the number of hospitals per 10,000 elderly population, the number of beds of long-term care facilities per 10,000 elderly population, total population (10,000 persons), average per capita annual income (million yen) , percentage of elderly people (%), and percentage of single-person households with elderly people (%)

a GWR settings:

Model type: Gaussian

Geographic kernel: adaptive bi-square

Method for optimal bandwidth search: Golden section search

Criterion for optimal bandwidth: AICc

b Moran's I: 42 municipalities those with no neighbors were excluded.
